# The *Fusarium graminearum* Genome Reveals More Secondary Metabolite Gene Clusters and Hints of Horizontal Gene Transfer

**DOI:** 10.1371/journal.pone.0110311

**Published:** 2014-10-15

**Authors:** Christian M. K. Sieber, Wanseon Lee, Philip Wong, Martin Münsterkötter, Hans-Werner Mewes, Clemens Schmeitzl, Elisabeth Varga, Franz Berthiller, Gerhard Adam, Ulrich Güldener

**Affiliations:** 1 Institute of Bioinformatics and Systems Biology, Helmholtz Zentrum München, German Research Center for Environmental Health (GmbH), Neuherberg, Germany; 2 Wellcome Trust Centre for Human Genetics, University of Oxford, Oxford, United Kingdom; 3 Department of Chemical Engineering and Applied Chemistry, University of Toronto, Toronto, Canada; 4 Department of Genome Oriented Bioinformatics, Technische Universität München, Wissenschaftszentrum Weihenstephan, Freising, Germany; 5 Department of Applied Genetics and Cell Biology, University of Natural Resources and Life Sciences, Tulln, Austria; 6 Christian Doppler Laboratory for Mycotoxin Metabolism and Center for Analytical Chemistry, Department for Agrobiotechnology (IFA-Tulln), University of Natural Resources and Life Sciences, Tulln, Austria; University of Wisconsin - Madison, United States of America

## Abstract

Fungal secondary metabolite biosynthesis genes are of major interest due to the pharmacological properties of their products (like mycotoxins and antibiotics). The genome of the plant pathogenic fungus *Fusarium graminearum* codes for a large number of candidate enzymes involved in secondary metabolite biosynthesis. However, the chemical nature of most enzymatic products of proteins encoded by putative secondary metabolism biosynthetic genes is largely unknown. Based on our analysis we present 67 gene clusters with significant enrichment of predicted secondary metabolism related enzymatic functions. 20 gene clusters with unknown metabolites exhibit strong gene expression correlation *in planta* and presumably play a role in virulence. Furthermore, the identification of conserved and over-represented putative transcription factor binding sites serves as additional evidence for cluster co-regulation. Orthologous cluster search provided insight into the evolution of secondary metabolism clusters. Some clusters are characteristic for the *Fusarium* phylum while others show evidence of horizontal gene transfer as orthologs can be found in representatives of the *Botrytis* or *Cochliobolus* lineage. The presented candidate clusters provide valuable targets for experimental examination.

## Introduction

In fungal genomes, genes involved in specific as well as common metabolic pathways have been observed to form tightly linked clusters on the chromosomes [1]–[5]. Some of these clustered genes are of major interest and are intensively studied due to the pharmacological properties of the secondary metabolites (SM) resulting from the activities of the gene products. Examples are clusters required for the production of mycotoxins, such as aflatoxins, ochratoxins and trichothecenes, or clusters for the synthesis of antibiotics. Despite the potential importance concerning human health or economic impact, it is difficult to identify the chemical products associated with fungal gene clusters because many clustered genes are not expressed under laboratory conditions [6], [7]. Gene expression may only be observed during a specific stage during plant infection [8] or upon contact with another microbe [9], [10]. Manipulation of genes coding for specific transcription factors or proteins with a role in establishment or maintenance of specific heterochromatic chromatin structures may help by inducing gene expression *in vitro* [11]–[16]. With a rapidly increasing number of fully sequenced fungal genomes at hand, identification and analysis of tentative gene clusters using bioinformatic tools should foster functional analysis leading to discovery of new natural products. The comparative analysis of SM gene clusters in diverse genomes should give insight into their evolution and origin.

To identify fungal gene clusters, functional attributes of adjacent genes can be used as a starting point. This approach has already been conducted in diverse sequenced eukaryotic genomes by exploiting co-expression, or predicted common function. Microarray data were used for genome wide studies of gene expression in relation to gene order or protein function [17]–[19]. By measuring correlations among expression profiles of adjacent genes it was possible to find gene clusters involved in a common pathway. Genomic clustering of co-expressed fungal genes was first identified in *Saccharomyces cerevisiae* [20] and later observed in diverse eukaryotes [21]. Fungal secondary metabolite gene clusters are often species-specific and have diverse origins [22]–[24], potentially derived from horizontal gene transfer of clusters or conditionally dispensable chromosomes [4], [25].

To predict functional gene clusters, comparative genome analysis is a limited, but valuable approach as highlighted by the analysis of epipolythiodioxopiperazines (ETP), a class of secondary metabolite toxins produced by various ascomycetous fungi [26]. A member of the ETP gliotoxin cluster was identified in the animal pathogen *Aspergillus fumigatus* by homology search using genes from the ETP sirodesmin cluster of the plant pathogen *Leptosphaeria maculans* [27], [28]. This case is an example showing that known gene clusters may allow identification of related gene clusters in other fungal genomes.

As a major pathogen of cultivated cereals, *F. graminearum* was chosen as a target organism for the analysis of gene clusters at the genomic level in this study. In Table 1 we have summarized SM genes or gene clusters of *F. graminearum* for which the corresponding metabolites are already known. Yet, the genes with known functions (13 SM genes) cover only a minor fraction of the 51 predicted SM genes in *F. graminearum*: 15 polyketide synthetases (PKS), 19 nonribosomal peptide synthetases (NPS) and 17 terpenoid synthetases (TPS) although the numbers keeps changing over time [29]–[32]. These types of SM genes encode signature enzymes that can be enriched in secondary metabolism gene clusters and responsible for main synthesis steps of metabolites. The majority of the predicted SM genes have still unknown functions, but can serve as valuable entry points to search for functional gene clusters in the vicinity of those genes (Table S1). Besides the classical SM genes (TPS, NPS and PKS) the 114 predicted genes encoding cytochrome P450 enzymes (CYP) are also suitable candidates for searching secondary metabolite gene clusters. Cytochrome P450s play an essential role in many known biosynthetic pathways of fungal compounds, for instance in the biosynthesis of trichothecene mycotoxins [33] and gibberellins [34]. Further pathway steps responsible for modifications of the metabolites can involve tailoring enzymes such as methyltransferases, acyltransferases, oxidoreductases or glycosyltransferases. For the regulation of the metabolite production and export of synthesized compounds, transcription factors and transporter encoding genes are often co-localized in secondary metabolism clusters.

**Table 1:** Secondary metabolites produced by *F. graminearum* and corresponding biosynthetic genes or gene clusters.

Cluster ID	Metabolite	Gene range, (Number of genes)	Key enzyme	Pharmacological property (as mycotoxin), Role in plant pathogenesis	References
C49	Butenolide	FGSG_08077 ∼ FGSG_08084, (8)	*CYP*	Low oral toxicity, depletes glutathione, no significant effect of gene disruption.	[50], [105] www.scabusa.org/pdfs/forum06_proc_pgg
C28	Carotenoid	FGSG_16340	*DTC1*	Terpenoid pigment	[106]
C63	Malonichrome	FGSG_11026	*NPS1*	Extracellular siderophor, induced *in planta*.	[107], Berthiller et al. in prep.
C33	Ferricrocin	FGSG_05372	*NPS2*	Intracellular siderophore.	[108]
C21	Triacetylfusarinine	FGSG_03747	*NPS6*	Main extracellular siderophore, conserved role in virulence.	[109]
C53	n.d.	FGSG_17168	*PKS3*	Precursor of insoluble perithecial pigment.	[110]
C15	Zearalenone	FGSG_17745 and FGSG_15980	*PKS4, PKS13*	Powerful xenoestrogen in animals, no effect on virulence.	[68], [72]
C60	Fusarielin	FGSG_10455- FGSG_10465, (7)	*PKS9*		[54]
C42	Fusarin C	FGSG_07798	*PKS10*	Possible carcinogen, mutagen, instable compound.	[51]–[53], [111]
C13	Aurofusarin Rubrofusarin	FGSG_02320 ∼ FGSG_02329, (10)	*PKS12*	Golden yellow/red pigment of mycelium, low toxicity (high concentrations in feed can affect antioxidant levels in eggs).	[36], [112]–[115]
C18	Orcinol	FGSG_03971 - FGSG_03956, (18)	*PKS28*	Responsible for production of orsellinic acid/orcinol.	[116]
C59	Culmorin	FGSG_10397	*TPS*	Antifungal, phytotoxic in high concentrations.	[117]
C23	Trichothecene	FGSG_03543 ∼ FGSG_03532, (12)	*TRI5*	Protein biosynthesis inhibitor, virulence factor on wheat.	[48]

Currently known secondary metabolites of *F. graminearum* and corresponding genes (gene clusters) required for biosynthesis. PKS: Polyketide synthases, NPS: Non-ribosomal peptide synthetase, TPS: Terpenoid synthases, CYP: Cytochrome P450.

The *de novo* prediction tool SMURF [18] utilizes this characteristic functional composition to predict gene clusters based on protein domains. The application of the method on the *F. graminearum* genome elucidated many putative, but also known gene clusters and demonstrated the efficiency of domain based *de novo* prediction methods. A similar approach with a focus on PKS and TPS clusters has been performed by Ma et al. (Ma et al., 2010). 15 novel clusters have been predicted using functional domain information in combination with two microarray experiments of expression quantification during plant infection and sexual development as evidence. This set of predicted clusters was extended with four novel clusters that were identified based on co-expression analysis by Zhang et al. using time series microarray experiments of *F. graminearum* growing inside wheat coleoptiles (Zhang et al., 2012). Utilizing four microarray experiments as co-expression evidence, Lawler et al. showed that co-expressed cluster genes in *F. graminearum* often contain transcription associated proteins such as transcription factors and genes involved in biosynthetic pathways like the butenolide gene cluster [19].

In this work we present a *de novo* approach that utilizes four sources of evidence to predict novel gene clusters and to validate known ones (Table 1). We predicted candidate PKS, NPS and TPS clusters based on functional domain composition and identified over-represented promoter motifs which suggest co-regulation. We determined evolutionary conservation of gene clusters by searching a protein similarity database of 332 completely sequenced genomes for orthologous clusters. Finally we analysed 12 microarray experiments in order to determine co-expression of genes with an emphasis on expression during plant infection (Table 2). Besides 12 known key enzymes/clusters, our analyses identified 55 putative SM gene clusters (Table S2). Remarkably; additional genes which may be part of three known gene clusters were found (trichothecene, malonichrome and triacetylfusarinin), provoking further analysis of these functional modules.

**Table 2:** Used gene expression experiments.

*PlexDB Number*	*Experiment Name*	*Reference*
FG1	*Fusarium* transcript detection on Morex barley spikes using *Fusarium* Affy GeneChips	[38]
FG2	Expression Profiles in Carbon and Nitrogen Starvation Conditions	[38]
FG7	*Fusarium* gene expression profiles during conidia germination stages	[39]
FG10	Response to trichodiene treatment in *Fusarium graminearum*	[43]
FG11	Gene Regulation by *Fusarium* Transcription Factors Tri6 and Tri10	[43]
FG12	*Fusarium graminearum* gene expression during crown rot of wheat	[42]
FG13	The transcription factor FgStuAp influences spore development, pathogenicity and secondary metabolism in *Fusarium graminearum*	[41]
FG14	DON induction media	[55]
FG15	*Fusarium graminearum* gene expression during wheat head blight	[41]
FG16	*Fusarium graminearum* gene expression in wheat stems during infection	[47]
FG18	Trichothecene synthesis in a *Fusarium graminearum* Fgp1 mutant	[45]
FG19	Stage-specific expression patterns of *Fusarium graminearum* growing inside wheat coleoptiles with laser microdissection	[40]

Overview of used gene expression experiments, data obtained from PlexDB [37].

## Results

### Screening neighboring genes for functional gene clusters

**Figure 1:**
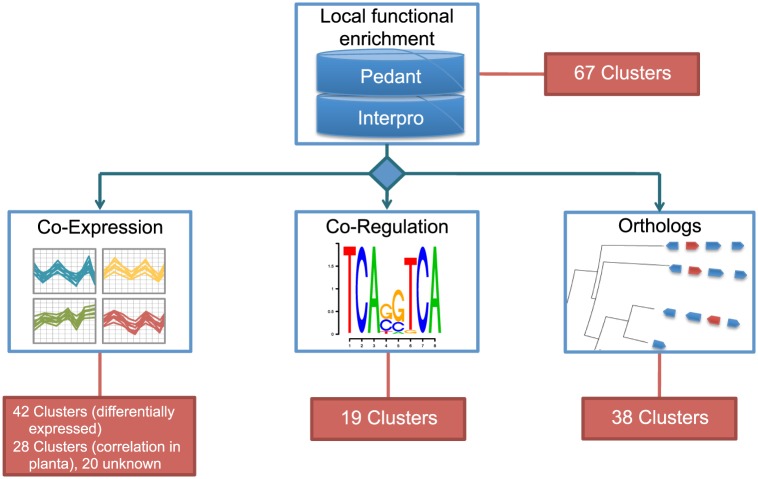
Workflow for the prediction of gene clusters in F. graminearum. Based on the gene functions found in known fungal gene clusters, 67 gene clusters with specific collections of gene functions were identified by screening the F. graminearum scaffolds. Expression data analysis resulted in 43 clusters that are either differentially expressed in at least one condition or show correlation in expression profiles during plant infection. Conserved promoter motifs could be computed in 19 clusters. 38 Clusters have orthologs in organisms outside the Fusarium clade.

Based on the compositions of experimentally elucidated clusters we scanned for local accumulations of SM signature genes (TPS, PKS, NPS, DMATS (dimethylallyltryptophan synthases)) and tailoring enzyme genes (methyltransferases, acyltransferases, oxidoreductases, glycosyltransferases and cytochrome P450s) and performed a functional enrichment analysis of secondary metabolism related functions to determine the significance of the gene clusters. A total number of 67 statistically significant (P-value <0.05, Fisher's exact test [35]) potential gene clusters presumably involved in secondary metabolite biosynthesis were identified in this way (Figure 1, Table S2). A functional domain-based prediction of putative SM genes revealed 15 PKS, 23 NPS/NPS-like, 17 TPS and 114 P450 genes. We did not find DMATS genes in *F. graminearum*. Besides tailoring enzymes, 40 clusters contain at least one predicted signature enzyme. The clusters contain about 58% (15 PKS, 21 NPS, 14 TPS, 48 P450) of the predicted SM genes. In particular, the genes with known functions from metabolite clusters reported for *F. graminearum* in Table 1 are all included in these clusters. Some of the predicted clusters represent extensions of functional gene clusters. For example ten genes (FGSG_02320 - FGSG_02329) are involved in the synthesis of aurofusarin [36]. However, the neighboring laccase precursor related gene (FGSG_02330) correlates in gene expression with the aurofusarin genes and thus is included in cluster C13.

### 42 predicted clusters are supported by expression data

In many cases the genes of secondary metabolism gene clusters cover a whole secondary metabolism pathway. The genes can be co-regulated depending on the respective environmental conditions like the gibberellin cluster in *Fusarium fujikuroi* which is expressed under nitrogen starving conditions [5]. Examining the expression profiles of cluster genes can help to identify the environmental factors that are necessary for the metabolite production and uncover additional neighboring genes that are potentially part of the functional gene clusters.

The co-expression of neighboring genes was explored using twelve microarray datasets obtained from PlexDB [37]. The data comprises five time series experiments (Table 2) measuring gene expression during plant infection or different conditions [38]–[42] and seven case control studies investigating the effects of transcription factor deletions [43]–[45], the impact of different growth conditions [38], [43], [46] and the expression profile of different stages during infection of wheat stems and perithecia production [47] (Table 2).

**Figure 2:**
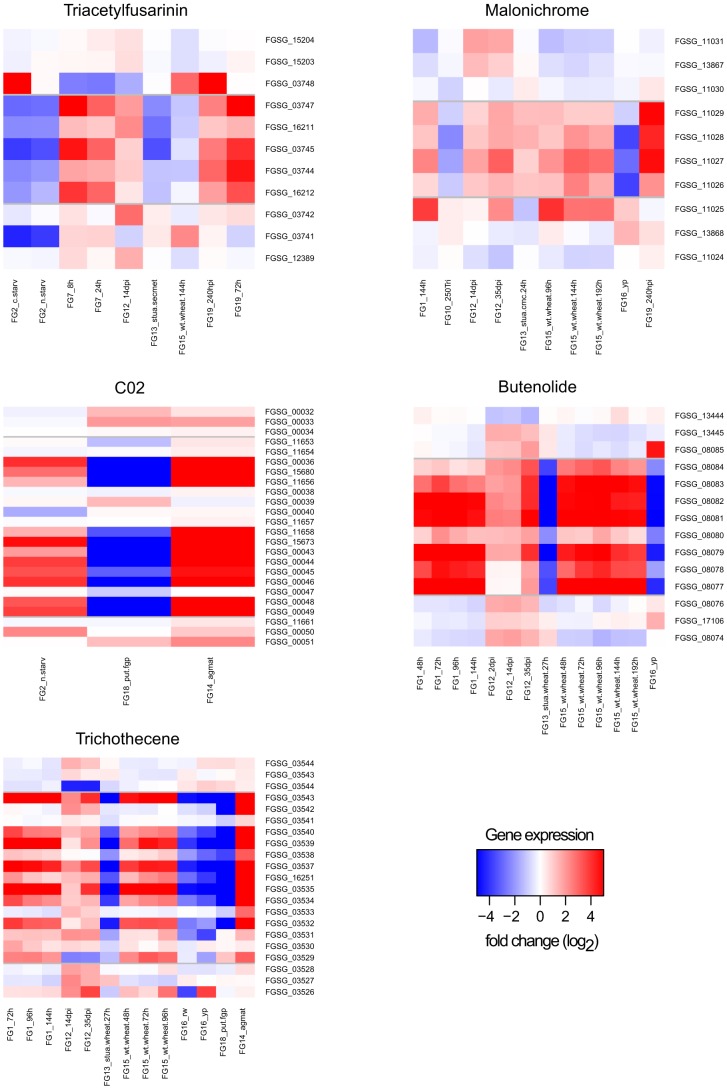
Differential gene expression heatmap of clusters and neighboring genes. Heatmaps illustrate fold changes in gene expression (log2 scale) between experimental conditions. Genes are listed in chromosomal order on y-axis. Abbreviations of experimental conditions on x-axis are according to Table 6. Horizontal grey bars show boundaries of predicted clusters.

We found 42 clusters with more than 60% of genes differentially expressed in at least one condition (see Methods). In 28 out of the 42 clusters we could determine a significant correlation in the expression profile in at least one of the time series experiments. The 28 clusters include the known gene clusters of the metabolites trichothecene [48], [49], butenolide [50], fusarin C [51]–[53], fusarielin [54] and aurofusarin [36]. Besides these known, experimentally validated pathways we found correlations in gene expression in the neighboring genes of the biosynthetic enzymes of triacetylfusarinin and malonichrome. Five genes in a cluster with enzymes involved in triacetylfusarinin biosynthesis show differential expression and correlation in their expression profile during infection. Interestingly all genes (FGSG_03747 to FGSG_16212) are significantly down-regulated (absolute fold change on log2 scale (|log2-FC|) above 1.4, P-value below 0.05) during C- and N- starvation conditions (FG2) but up-regulated during conidiation (FG7) and infection of wheat (FG19) (Figure 2).

Four neighboring genes of the malonichrome associated *NPS1* (FGSG_11026) are significantly up-regulated (|log2-FC| >1, P-value <0.05) during the infection process of wheat and exhibit correlated expression profiles during barley and wheat infection. Interestingly the genes are down-regulated when forming perithecia (FG16) and during trichodiene treatment (FG10) (Figure 2). The promoter analysis resulted in a significantly enriched motif CAGGGATCGGCC (P-value  =  9.17e-6), which is present in the promoters of the genes FGSG_11029 to FGSG_11026, but not in the promoter of the transcription factor FGSG_11025. The pathway genes of both siderophores (triacetylfusarinine, malonichrome) in *F. graminearum* are not experimentally determined yet. Our results give a hint on the borders of the gene cluster.

### Predicted secondary metabolism clusters exhibit characteristic gene expression *in planta*

To select predicted clusters that play a role during host infection, we focus on the gene expression measurements of experiments *in planta*. The time series data spans the first hours after infection up to several days. We calculated the Pearson correlation coefficient of neighboring genes and found correlations of gene expression profiles in 28 clusters which are above the 95^th^ percentile of randomly sampled genes of the genome. Beside the known synthesis genes of aurofusarin, zearalenone, trichothecenes, butenolide, triacylfusarinin and malonichrome we determined correlations in 20 predicted clusters of which the associated metabolite is unknown.

**Figure 3:**
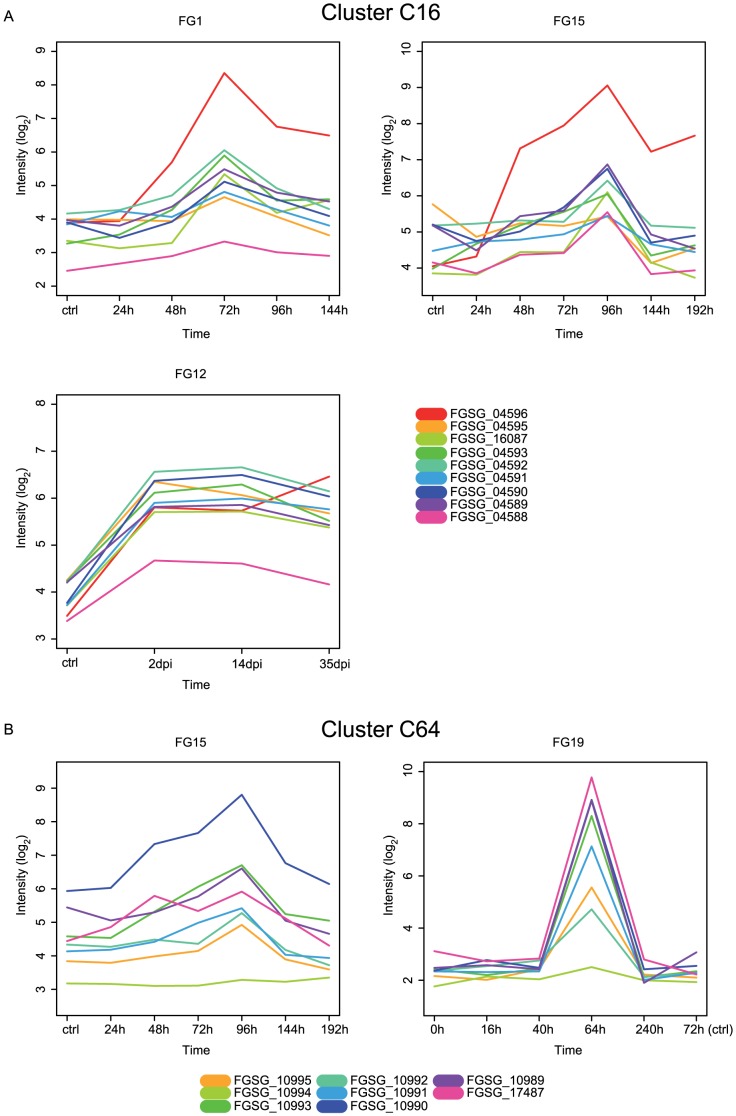
Expression profiles of gene cluster C16 and C64 during *in planta* time series experiments. Expression profiles of gene cluster C16 and C64. Time points are depicted on x-axis, y-axis shows log2 intensity of gene expression. (A) Expression profile of cluster C16 during barley (FG1) and wheat infection (FG12, FG15). (B) Gene expression during wheat infection (FG15, FG19) of cluster C64.

The expression profiles of cluster C16 which contains *PKS29* (FGSG_04588), a terpenoid synthetase and two methyltransferases is significantly increased after 72 h post inoculation (hpi) on barley (FG1, Figure 3A) [38]. During the infection process of wheat, the expression of genes increases significantly after 96 h and decreases afterwards (FG15, Figure 3A) [41]. However in a second experiment, gene expression after 35 days post inoculation is still increased compared to the control measurement in complete defined media [42] (FG12, Figure 3A, Table 3).

**Table 3:** Functional description of cluster genes with correlated expression pattern.

*Cluster ID*	*Position*	*Gene Code*	*Description*
C16	1	FGSG_04596	related to O-methyltransferase
	2	FGSG_04595	related to hydroxylase
	3	FGSG_16087	hypothetical protein
	4	FGSG_16088	related to 3-ketoacyl-acyl carrier protein reductase
	5	FGSG_04593	related to para-hydroxybenzoate polyprenyltransferase precursor
	6	FGSG_04592	related to light induced alcohol dehydrogenase Bli-4
	7	FGSG_04591	probable farnesyltranstransferase (al-3)
	8	FGSG_04590	related to isotrichodermin C-15 hydroxylase (cytochrome P-450 monooxygenase CYP65A1)
	9	FGSG_04589	related to tetracenomycin polyketide synthesis O-methyltransferase tcmP
	10	FGSG_04588	polyketide synthase
C64	1	FGSG_10996	conserved hypothetical protein
	2	FGSG_10995	related to multidrug resistance protein
	3	FGSG_10994	conserved hypothetical protein
	4	FGSG_10993	related to selenocysteine lyase
	5	FGSG_10992	related to polysaccharide deacetylase
	6	FGSG_10991	related to benzoate 4-monooxygenase cytochrome P450
	7	FGSG_10990	related to AM-toxin synthetase (AMT)
	8	FGSG_10989	conserved hypothetical protein
	9	FGSG_17487	related to non-ribosomal peptide synthetase

Functional gene descriptions of predicted clusters C16 and C64 illustrated in Figure 3.

Like the gene expression profile of the aurofusarin cluster genes which shows a peak after 64 hpi on wheat seedling coleoptiles, the cluster C64 show a very similar profile (FG19, Figure 3B) [40]. The cluster consists of nine genes including two NPS key enzymes and one cytochrome P450 protein. All genes are up-regulated simultaneously after 64 h and down regulated afterwards as already reported by Zhang et al. 2012. Beyond that, an increase in gene expression can also be observed during the infection of barley [42] where the maximum expression is reached after 96 hpi [41] (Figure 3B, Table 3).

These results show that the genes of predicted clusters can be co-expressed *in planta* and show similar expression profiles like those of the trichothecene or aurofusarin cluster genes. It is likely that these clusters code for novel metabolites which have an impact in plant pathogenesis and are targets for further experimental investigation.

### Palindromic promoter motif correlates with gene expression

To help determine which clusters are regulated by a secondary metabolism specific transcription factor, we scanned the promoter regions of each cluster for conserved binding motifs. We applied Fisher's exact test to determine the significance of motifs found in cluster gene promoters compared to the genome wide distribution of the motifs. In total we identified 19 clusters which contain a significantly over-represented promoter motif (P-value <0.01, Table S2). One of the most significant motifs is the palindrome 5′-GTGGtgCCAC-3′ in the cluster C02 as previously reported [55] (Table 4). The cluster contains 16 genes (FGSG_11653 - FGSG_00049) of which 12 genes carry the putative palindromic binding site in their promoters (Table 4). As already reported, the gene expression of 11 of the 12 putative target genes is significantly increased in the wild-type when growing on agmatine- compared to glutamine- medium (4.6 to 9.2 fold on log2 scale, P-value <0.05) [55]. Interestingly, the expression of all 12 genes is significantly lower in the mutant of the *FGP1* transcription factor while growing on putrescine-medium (1 to 8.5 fold on log2 scale, P-value <0.05) [45]. Additionally under nitrogen starving conditions a significant increase in gene expression in seven genes takes place (3.4 to 4.8 fold on log2 scale, P-value <0.05) [38] (Figure 1). The motif occurs in only 4% of all promoters on the genome and is significantly enriched in this cluster (P-value  =  6.8e-13).

**Table 4:** Functional description of co-expressed cluster genes.

*Cluster ID*	*Position*	*Gene_Code*	*Description*	*Predicted Motif*
Triacetylfusarinin	−3	FGSG_15204	hypothetical protein	
	−2	FGSG_15203	hypothetical protein	
	−1	FGSG_03748	conserved hypothetical protein	
	1	FGSG_03747	related to AM-toxin synthetase (AMT)	
	2	FGSG_03745	related to aerobactin siderophore biosynthesis protein iucB	
	3	FGSG_03744	related to major facilitator MirA	
	4	FGSG_03742	related to cellobiose dehydrogenase	
	5	FGSG_03741	related to O-methylsterigmatocystin oxidoreductase	
	+1	FGSG_12389	conserved hypothetical protein	
	+2	FGSG_16211	related to enoyl-CoA hydratase	
	+3	FGSG_16212	hypothetical protein	
Malonichrome	−3	FGSG_11031	hypothetical protein	
	−2	FGSG_13867	hypothetical protein	
	−1	FGSG_11030	related to ferric reductase Fre2p	
	1	FGSG_11029	related to major facilitator MirA	TAGGGATCGGCG
	2	FGSG_11028	related to ATP-binding cassette transporter protein YOR1	CAGGGATCGGCC
	3	FGSG_11027	conserved hypothetical protein	CAGGGATCGGCC
	4	FGSG_11026	non-ribosomal peptide synthetase	CAGGGATCGGCA
	5	FGSG_11025	putative C2H2 zinc finger transcription factor	
	+1	FGSG_13868	conserved hypothetical protein	
	+2	FGSG_11024	probable cytochrome P450 51 (eburicol 14 alpha-demethylase)	
	+3	FGSG_11023	conserved hypothetical protein	
C02	−3	FGSG_00032	related to non-heme chloroperoxidase	
	−2	FGSG_00033	conserved hypothetical protein	
	−1	FGSG_00034	related to alpha-glucoside transport protein	
	1	FGSG_11653	probable sulfatase	
	2	FGSG_11654	related to nitrate assimilation regulatory protein	
	3	FGSG_00036	probable fatty acid synthase, alpha subunit	GTGGtgCCAC
	4	FGSG_11656	related to FAS1 - fatty-acyl-CoA synthase, beta chain	GTGGtgCCAC
	5	FGSG_00038	hypothetical protein	GTGGtgCCAC
	6	FGSG_00039	conserved hypothetical protein	
	7	FGSG_00040	conserved hypothetical protein	
	8	FGSG_11657	conserved hypothetical protein	
	9	FGSG_11658	hypothetical protein	
	10	FGSG_00043	conserved hypothetical protein	
	11	FGSG_00044	conserved hypothetical protein	GTGGtgCCAC
	12	FGSG_00045	conserved hypothetical protein	GTGGtgCCAC
	13	FGSG_00046	related to multidrug resistance protein	GTGGtgCCAC
	14	FGSG_00047	conserved hypothetical protein	GTGGtgCCAC
	15	FGSG_00048	related to flavonol synthase-like protein	GTGGtgCCAC
	16	FGSG_00049	related to branched-chain amino acid aminotransferase	GTGGtaCCAC
	17	FGSG_11661	conserved hypothetical protein	GTGGtgCCAC
	18	FGSG_00050	conserved hypothetical protein	GTGGtgCCAC
	+1	FGSG_00051	related to aliphatic nitrilase	
	+2	FGSG_15673	non-ribosomal peptide synthetase	
	+3	FGSG_15680	related to benzoate-para-hydroxylase (cytochrome P450)	
Butenolide	−3	FGSG_13444	related to allantoate transporter	
	−2	FGSG_13445	probable benzoate 4-monooxygenase cytochrome P450	
	−1	FGSG_08085	conserved hypothetical protein	
	1	FGSG_08084	related to monocarboxylate transporter 4	TAATGCTCCG
	2	FGSG_08083	related to glutamic acid decarboxylase	AAATGGACCG
	3	FGSG_08082	conserved hypothetical protein	AAATGGACCG
	4	FGSG_08081	related to gibberellin 20-oxidase	AAATTGTCCG
	5	FGSG_08080	conserved hypothetical protein	AAGTGCTCCG
	6	FGSG_08079	probable benzoate 4-monooxygenase cytochrome P450	TAATGCTCCG
	7	FGSG_08078	related to general amidase	AAATGCTCCG
	8	FGSG_08077	related to flavin oxidoreductase	AAATGCTCCG
	+1	FGSG_08076	hypothetical protein	
	+2	FGSG_17106	hypothetical protein	
	+3	FGSG_08074	conserved hypothetical protein	
Trichothecenes	−3	FGSG_03545	related to OrfH - unknown, trichothecene gene cluster	
	−2	FGSG_12416	conserved hypothetical protein	
	−1	FGSG_03544	deacetylase	
	1	FGSG_03543	putative trichothecene biosynthesis gene	TCAGGCCT
	2	FGSG_03542	probable cytochrome P450	
	3	FGSG_03541	trichothecene efflux pump	TCAGGCCT
	4	FGSG_03540	isotrichodermin C-15 hydroxylase	TTAGGCCT
	5	FGSG_03539	hypothetical protein	TCAGGCCT
	6	FGSG_03538	regulatory protein	
	7	FGSG_03537	trichodiene synthase [sesquiterpene cyclase]	TAAGGCCT
	8	FGSG_16251	trichothecene biosynthesis positive transcription factor	TCAGGCCT
	9	FGSG_03535	trichodiene oxygenase [cytochrome P450]	TCAGGCCT
	10	FGSG_03534	trichothecene 15-O-acetyltransferase	
	11	FGSG_03533	related to TRI7 - trichothecene biosynthesis gene cluster	TCAGGCCT
	12	FGSG_03532	trichothecene 3-O-esterase	TCAGGCCT
	13	FGSG_03531	monooxygenase	
	14	FGSG_03530	acetylesterase, trichothecene gene cluster	TCAGGCCT
	15	FGSG_03529	related to glucan 1,3-beta-glucosidase	TCAGGCCT
	+1	FGSG_03528	conserved hypothetical protein	
	+2	FGSG_03527	conserved hypothetical protein	
	+3	FGSG_03526	unknown, trichothecene gene cluster	

Functional gene descriptions and positions of over-represented promoter motifs on predicted clusters and neighboring genes. Expression of genes is illustrated in Figure 2.

The experimentally elucidated genes of the butenolide cluster [50] exhibit significant differential expression *in planta* (FG1, FG12 and FG15) whereas the neighboring genes do not correlate significantly in expression (Figure 1). We identified the significantly enriched binding motif 5′-[AT] A [AG] T [GT] [CG] [TA] CCG-3′ in all of the differentially expressed genes (Table 4).

Cluster specific putative binding sites could also be found in gene clusters for known metabolites like fusarin C, malonichrome and trichothecenes (see Methods). In the case of the trichothecene cluster the identified promoter motif matches the known binding site of the orthologous genes in *F. sporotrichoides* [56]. The results hint towards a specific regulation by transcription factors of these 19 putative clusters.

### The trichothecene gene cluster – larger than assumed?

12 genes are currently referred to as the core trichothecene gene cluster as a result of gene deletion or disruption experiments in *F. graminearum* and *F. sporotrichioides* [48]. Additional genes may be required for trichothecene biosynthesis because a few steps leading to the end products of the trichothecene biosynthetic pathway such as T-2 toxin and deoxynivalenol (DON) are still unknown [57], [58]. In the case of T-2 toxin biosynthesis by *F. sporotrichioides* it is unknown how the iso-valeryl-group is generated from leucine. In the case of DON it is unknown how *F. graminearum* converts the C8-OH into a keto group. There are three additional genes occurring in the gene cluster based on co-expression, downstream of *TRI8* (FGSG_03531 - FGSG_03529) (Figure 2). The motif seed 5′-TnAGGCCT-3′ in this cluster is significantly enriched (P-value  = 0.0042) in the putative promoters of 11 genes (Table 4, FGSG_03543 (TRI14) - FGSG_03529) and appears between the second and third additional gene. This motif seed is identical with the DNA-binding site 5′-TnAGGCCT-3′ previously established for the Cys2His2 zinc-finger regulatory protein TRI6, a positive regulator of trichothecene biosynthesis in *F. sporotrichioides* [56]. However, others have experimentally determined the binding affinity of TRI6 to a different motif [59], which is located in five of the cluster gene promoters, but the motif could not be detected by our approach. The other striking evidence for the presence of these three additional genes in the cluster arises in the results from co-expression data. (Figure 1 and Table 4) [38], [41]. All the genes in the trichothecene biosynthesis cluster including the three additional genes are co-expressed with an increasing expression pattern until the third day of growth except for three genes, cytochrome P450 (FGSG_03542), FGSG_03541 and FGSG_03533. This observation strongly supports the assumption that the new cluster genes might be involved in yet unknown steps of the trichothecene biosynthetic pathway or a trichothecene related function. DON is glycosylated *in planta* [60], which inactivates the toxin. *F. graminearum* has β-glucosidase activity, which efficiently hydrolyzes the glucoside and restores the active toxin (data not shown). Recently fungal 1,3-beta-glucanases have been shown to possess this activity [61]. Since one of the co-regulated genes (FGSG_03529) next to the core TRI cluster is annotated as “related to glucan 1,3-beta-glucosidase” we have tested the hypothesis that this gene may encode an enzyme reactivating plant-neutralized DON. To this end we expressed a cDNA version (for primers and construction details see Methods) in the host *Saccharomyces cerevisiae*, which is devoid of DON-3-glucoside (D3G) hydrolytic activity. Yet, using HPLC-MS no D3G hydrolytic activity of transformed yeast cells could be detected.

FGSG_03530 is annotated as “hypothetical protein similar to acetylesterase”. The Tri8 esterase removes the first acetyl-residue from the biosynthetic precursor 3,15-diacetyl-DON. Depending on which allele is present, either 3-acetyl-DON (3-ADON) or 15-acetyl-DON (15-ADON) is formed [62]. It is still unknown which activity later removes also the remaining acetyl-group in axenic cultures. Due to the annotation of FGSG_03530 we tested whether expression of this gene in yeast endows yeast with the ability to remove the acetyl group and produce DON if treated with either 3-ADON or 15-ADON. Yet, no esterase activity could be detected using HPLC-MS. Experimental details are also given in Methods.

### The C47/*PKS23* cluster ortholog in *Botrytis fuckeliana* shows evidence of horizontal gene cluster transfer

The gene cluster inventory in closely related fungal species can differ significantly [5]. Due to their locally clustered topology, genes of a secondary metabolism pathway can be acquired by other fungi in a single horizontal gene transfer event [24], [63], [64]. Using the similarity matrix of proteins (SIMAP, [65]), we applied a database query based on protein similarity to all predicted 67 clusters and determined 38 clusters to have an ortholog in other species outside the *Fusarium* phylum. In two cases all publicly available sequenced *Fusarium* species (*Fusarium pseudograminearum*, *F. fujikuroi*, *F. verticillioides*, *F. oxysporum*, *F. solani*) lack the whole cluster present in *F. graminearum* whereas the more distant related genomes *Botrytis fuckeliana*, *Cochliobolus heterostrophus* and *Pyrenophora teres* contain an orthologous cluster.

**Figure 4:**
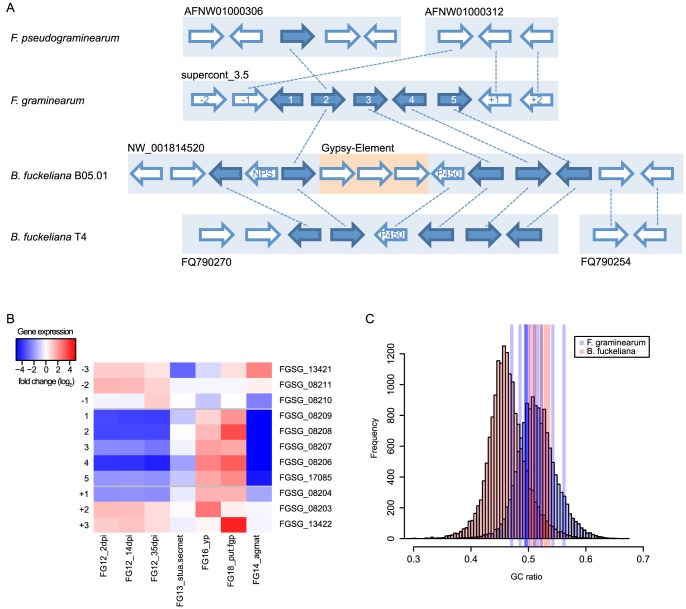
Evidence of horizontal gene transfer and regulation of predicted C47 cluster. (A) Predicted gene cluster in *Fusarium graminearum* and orthologous genes in *F. pseudograminearum* and the *Botrytis fuckeliana* strains B05.01 and T4 (solid dark blue arrows) on their respective supercontigs (light blue boxes). Adjacent genes are illustrated as white arrows, dashed lines depict orthologous groups. The gypsy transposable element in *B. fuckeliana* B05.01 is indicated as orange box. Enumeration in *F. graminearum* is according to Table 5. (B) The heatmap illustrates fold changes in gene expression (log2 scale) between two experimental conditions. Genes are listed in chromosomal order on y-axis. Abbreviations of experimental conditions on x-axis are according to Table 6. Horizontal grey bars show boundaries of predicted clusters. (C) Histograms show whole genome distributions of open reading frame GC ratios in *F. graminearum* (blue) and *B. fuckeliana* B05.01 (red). Vertical lines illustrate GC ratios of cluster genes.

We predicted a cluster (C47, FGSG_08209 - FGSG_17085) in the vicinity of the signature enzyme *PKS23* (FGSG_08208, FG3_20) which contains also a NPS, a methyl transferase and a cytochrome P450 enzyme. The genes are repressed simultaneously during the infection of wheat [42] compared to the expression rate on complete defined medium (2.1 to 4.4 on log2 scale, P-value <0.05). Further the influence of DON-inducing agmatine in growth medium causes also a significant decrease in gene expression of the whole cluster (4.5 to 6.7 on log2 scale, P-value <0.05) (Figure 4B) [55]. Neither the metabolite synthesized by this cluster nor its function are known so far. An ortholog of this cluster can be found in the two *Botrytis fuckeliana* strains B05.01 and T4 whereas the neighboring genes are not present in both genomes (Figure 4A and Table 5). All other inspected genomes lack an orthologous gene cluster. In the closely related *F. pseudograminearum* the PKS enzyme (FGSG_08208) is the only cluster member which is represented by an ortholog. Orthologs of the surrounding genes of the *F. graminearum* cluster constitute a collinear region on a different scaffold. Orthologous clusters in the two *Botrytis fuckeliana* strains contain an additional P450 gene (B05.01: BC1G_09046, T4: BofuT4_059840.1) that is not present in *F. graminearum* and a NPS-like enzyme that is unique for the B05.01 strain (BC1G_09041). Additionally, a Gypsy transposable element BOTY_I ([66], Repeatmasker SW-score: 40718), consisting of three open reading frames (ORFSs), could be identified by aligning the RepBase library on the genome (Figure 4A and Table 5).

**Table 5:** Functional description of genes with putative HGT.

*Cluster ID*	*Position*	*Gene Code*	*Description*	*Predicted Motif*
C47	−2	FGSG_08211	conserved hypothetical protein	
	−1	FGSG_08210	conserved hypothetical protein	
	1	FGSG_08209	non-ribosomal peptide synthetase	TAGGGACTTTGG
	2	FGSG_08208	polyketide synthase	TAGGGACTTTGG
	3	FGSG_08207	related to cytochrome P450 7B1	TAGGAACTATGG
	4	FGSG_08206	conserved hypothetical protein	TTGGGACTTTGG
	5	FGSG_17085	related to ornithine aminotransferase	TTGGGACTTTGG
	+1	FGSG_08204	conserved hypothetical protein	
	+2	FGSG_08203	conserved hypothetical protein	
C61	−1	FGSG_17385	hypothetical protein	
	1	FGSG_10542	conserved hypothetical protein	
	2	FGSG_13782	putative protein	
	3	FGSG_10543	hypothetical protein	
	4	FGSG_17386	related to non-ribosomal peptide synthetase	
	5	FGSG_10545	conserved hypothetical protein	
	6	FGSG_10546	hypothetical protein	
	7	FGSG_10547	related to multidrug resistance protein	
	8	FGSG_17387	probable type I polyketide synthase	
	+1	FGSG_10549	conserved hypothetical protein	
C62	−2	FGSG_10606	probable cytochrome-c peroxidase precursor	
	−1	FGSG_10607	hypothetical protein	
	1	FGSG_10608	conserved hypothetical protein	
	2	FGSG_10609	related to 6-hydroxy-d-nicotine oxidase	
	3	FGSG_17400	related to cytochrome P450 monooxygenase	
	4	FGSG_17401	hypothetical protein	
	5	FGSG_10611	related to 6-hydroxy-d-nicotine oxidase	
	6	FGSG_10612	related to salicylate hydroxylase	
	7	FGSG_10613	related to para-hydroxybenzoate polyprenyltransferase precursor	
	8	FGSG_10614	conserved hypothetical protein	
	9	FGSG_17402	probable beta-glucosidase precursor	
	10	FGSG_10616	related to vegetatible incompatibility protein HET-E-1	
	11	FGSG_10617	related to nonribosomal peptide synthetase MxcG	
	+1	FGSG_10618	hypothetical protein	

**Figure 5:**
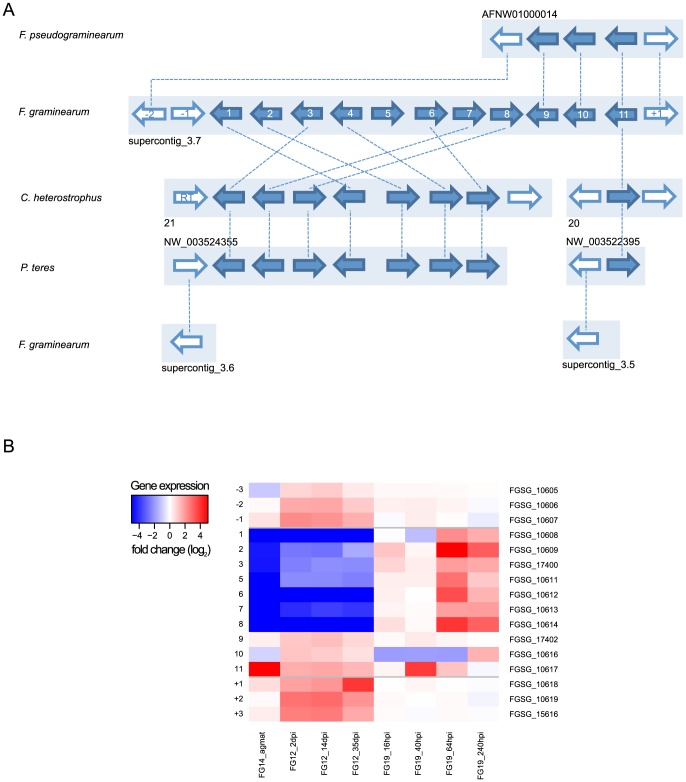
Orthologous genes and regulation of predicted C62 cluster. (A) Predicted gene cluster in *Fusarium graminearum* and orthologous genes in *F. pseudograminearum*, *Cochliobolus heterostrophus* and *Pyrenophora teres*. (solid dark blue arrows) on their respective supercontigs (light blue boxes). Adjacent genes are shown in white, dashed lines between genes illustrate orthologous groups. Enumeration in *F. graminearum* is according to Table 5. Reverse transcriptase in *C. heterostrophus* is indicated as “RT”. (B) Heatmap illustrates fold changes in gene expression (log2 scale) of cluster and adjacent genes between experimental conditions. Genes are listed in chromosomal order on y-axis. Horizontal grey bars show boundaries of predicted clusters. Abbreviations of experimental conditions on x-axis are according to Table 6. No expression data is available for FGSG_17401, as a distinct mapping of probes on this gene model was not possible.

**Figure 6:**
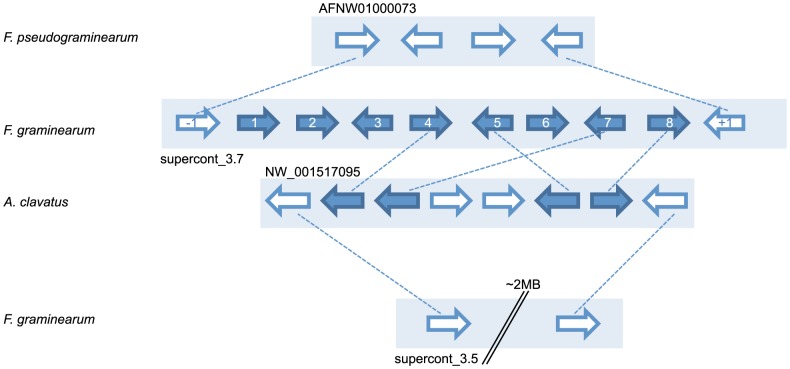
Orthologous genes of predicted C61 cluster. Predicted gene cluster in *Fusarium graminearum* and orthologous genes in *F. pseudograminearum* and *Aspergillus clavatus* are depicted in solid dark blue colors, adjacent genes are shown in white. Dashed lines illustrate orthologous groups. Enumeration in *F. graminearum* is according to Table 5.

Functional gene descriptions and positions of over-represented promoter motifs on predicted clusters and neighboring genes. Orthologs of the predicted clusters are shown in Figures 4, 5 and 6.

**Table 6:** Details of the used microarray data sets on conditions and strains.

*Condition Abbrevation*	*Case Condition*	*Control Condition*	*PlexDB Accession-No*	*Both_conditions*
FG1_24 h	Barley infection (24 h)	Water control	FG1	FG1_24 h.FG1_water
FG1_48 h	Barley infection (48 h)	Water control	FG1	FG1_48 h.FG1_water
FG1_72 h	Barley infection (72 h)	Water control	FG1	FG1_72 h.FG1_water
FG1_96 h	Barley infection (96 h)	Water control	FG1	FG1_96 h.FG1_water
FG1_144 h	Barley infection (144 h)	Water control	FG1	FG1_144 h.FG1_water
FG2_c.starv	C nutrient deficient medium	Complete medium	FG2	FG2_c.starv.FG2_complete
FG2_n.starv	N nutrient deficient medium	Complete medium	FG2	FG2_n.starv.FG2_complete
FG7_2 h	Conidiation (2 h)	Conidiation (0 h)	FG7	FG7_2 h.FG7_0 h
FG7_8 h	Conidiation (8 h)	Conidiation (0 h)	FG7	FG7_8 h.FG7_0 h
FG7_24 h	Conidiation (24 h)	Conidiation (0 h)	FG7	FG7_24 h.FG7_0 h
FG10_250Tri	Trichodiene medium	Normal medium	FG10	FG10_250Tri.FG10_0Tri
FG11_tri6	Tri6 deletion mutant	Wildtype	FG11	FG11_tri6.FG11_wt
FG11_tri10	Tri10 deletion mutant	Wildtype	FG11	FG11_tri10.FG11_wt
FG12_2dpi	Wheat infection (2 d)	Complete medium	FG12	FG12_2dpi.FG12_myc.cult
FG12_14dpi	Wheat infection (14 d)	Complete medium	FG12	FG12_14dpi.FG12_myc.cult
FG12_35dpi	Wheat infection (35 d)	Complete medium	FG12	FG12_35dpi.FG12_myc.cult
FG13_stua.cmc.24 h	FgStuA deletion mutant during spore production (24 h)	Wildtype during spore production	FG13	FG13_stua.cmc.24h.FG13_wt.cmc24h
FG13_stua.wheat.72 h	FgStuA deletion mutant during wheat infection (72 h)	Wildtype during wheat infection (72 h)	FG13	FG13_stua.wheat.72h.FG13_wt.fg13.72 h
FG13_stua.secmet	FgStuA deletion mutant during secondary metabolism inducing conditions	Wildtype during secondary metabolism inducing conditions	FG13	FG13_stua.secmet.FG13_wt.secmet
FG14_agmat	Agmatine medium (DON inducing)	Glutamine medium (DON non-inducing)	FG14	FG14_agmat.FG14_gln
FG15_wt.wheat.24 h	Wheat infection (24 h)	Water control	FG15	FG15_wt.wheat.24 h.FG15_ctrl.wheat
FG15_wt.wheat.48 h	Wheat infection (48 h)	Water control	FG15	FG15_wt.wheat.48 h.FG15_ctrl.wheat
FG15_wt.wheat.72 h	Wheat infection (72 h)	Water control	FG15	FG15_wt.wheat.72 h.FG15_ctrl.wheat
FG15_wt.wheat.96 h	Wheat infection (96 h)	Water control	FG15	FG15_wt.wheat.96 h.FG15_ctrl.wheat
FG15_wt.wheat.144 h	Wheat infection (144 h)	Water control	FG15	FG15_wt.wheat.144 h.FG15_ctrl.wheat
FG15_wt.wheat.192 h	Wheat infection (192 h)	Water control	FG15	FG15_wt.wheat.192 h.FG15_ctrl.wheat
FG16_rw	Radial growth	Infection front	FG16	FG16_rw.FG16_if
FG16_sw	Senescent wheat	Infection front	FG16	FG16_sw.FG16_if
FG16_yp	Perithecium formation	Infection front	FG16	FG16_yp.FG16_if
FG18_put.fgp	Fgp1 deletion mutant on putrescine medium	Wildtype on putrescine medium	FG18	FG18_put.fgp.FG18_put.wt
FG19_16hpi	Wheat infection (16 h)	Wheat infection (0)	FG19	FG19_16hpi.FG19_0hpi
FG19_40hpi	Wheat infection (40 h)	Wheat infection (0)	FG19	FG19_40hpi.FG19_0hpi
FG19_64hpi	Wheat infection (46 h)	Wheat infection (0)	FG19	FG19_64hpi.FG19_0hpi
FG19_240hpi	Wheat infection (240 h)	Wheat infection (0)	FG19	FG19_240hpi.FG19_0hpi

Experimental conditions and strains explored in expression data analysis. PlexDB accession numbers and abbrevations used in heatmaps (Figures 2, 4B and 5B) are given.

The GC-contents of the orthologous clusters are very similar (median GC content of 50.0% and 52.6% for *F. graminearum* and *B. fuckeliana* respectively) whereas the distributions of genome-wide GC-contents differ considerably (median GC content of 51.3% and 46.2% for *F. graminearum* and *B. fuckeliana*, respectively (Figure 4C)). We performed a two-sided Kolmogorov-Smirnov (KS) test for the GC distributions and obtained a significant (P-value  =  2.2e-16) difference between the GC-content of the *Botrytis* cluster genes and the genome-wide distribution of *Botrytis*. On the other hand the null hypothesis could not be rejected when comparing the GC-content of the same cluster ORFs to the genome-wide distribution of *F. graminearum* (P-value  = 0.4277). These results suggest a potential horizontal gene cluster transfer from the *Fusarium* lineage into *B. fuckeliana*.

Two clusters that could be linked to known metabolites show also hints of horizontal gene transfer (HGT). The genes of the metabolites aurofusarin and fusarielin are conserved in the closely related *F. pseudograminearum*, but cannot be found in other *Fusarium* species like the ones in the *Gibberella fujikuroi* species complex. Ten to seven genes of the aurofusarin cluster can be found in other species outside the *Fusarium* phylum. For example, the genes from FGSG_02320 to FGSG_02329 are conserved in *Trichophyton tonsurans*, but the orthologs of the PKS (FGSG_02324) and the adjacent gene (FGSG_02325) are located on another scaffold as the rest of the cluster. *Arthroderma benhamie* and *Arthroderma gypseum* have a syntenic cluster of eight genes, but totally lack orthologs of the PKS and the genes FGSG_02316 and FGSG_02321. In *A. gypseum* an ortholog of FGSG_02325 can be found on a different scaffold.

Further, the fusarielin cluster (C60) and its orthologs in *Aspergillus fumigatus*, *A. niger* and *A. clavatus* (as already described before [54]) were detected by our approach. The closely related *F. pseudograminearum* has seven of the eleven cluster genes, including the PKS (FGSG_10464) and the putative NPS (FGSG_10459) but lacking the cytochrome P450 enzyme (FGSG_10461).

### A NPS containing SM gene cluster shows expression during host infection and is conserved in *Cochliobolus heterostrophus* and *Pyrenophora teres*

In the peripheral region of chromosome one (at 267 kb) resides the putative cluster C62 (FGSG_10608 - FGSG_10617) consisting of eleven genes, containing a NPS and two cytochrome P450 genes. The core part of the cluster (FGSG_10608 - FGSG_10614) shows a co-expression pattern and is not present in the other *Fusaria*, but orthologs can be found in *Cochliobolus heterostrophus* and *Pyrenophora teres* (Figure 5A). The partially preserved cluster contains the two P450 genes and genes with FAD- and NAD(P)- binding domains. The NPS encoding gene is located on a separate contig and a reverse transcriptase can be found exclusively in *C. heterostrophus* next to the cluster. In order to test for a potential HGT-event we calculated the median ORF GC content of *C. heterostrophus* which is slightly higher compared to *F. graminearum* (53.4% vs. 51.3%). The GC contents of both clusters are in turn rather similar to each other (50.7% *C. heterostrophus*, 50.9% *F. graminearum*) and to the genome wide content of *F. graminearum*. However, when comparing the distributions of GC ratios of the cluster genes and the host genomes using a KS-test we calculated a significant difference in *C. heterostrophus* (P-value  = 0.002), but not in *P. teres* and *F. graminearum*. While taking a closer look at the gene expression during host infection, we observe that the NPS shows a significant increase in expression (3.8 fold on log2-scale, P-value <0.05) at 40 hpi while growing inside wheat coleoptiles. As mentioned before, this gene belongs not to the co-expressed core part of the cluster, consistent with insignificant change in expression of some of the other core genes at this time point. Yet, at 64 hpi the expression of the cluster genes that are conserved in *C. heterostrophus* and *P. teres* is significantly increased (1.9 to 5.1 fold on log2-scale, P-value <0.05) whereas the NPS is reduced [40] (Figure 5B and Table 5).

An opposite observation can be made when looking at the gene expression during infection of wheat stems where the gene expression of the core part is significantly down-regulated (1.7 to 6.4 fold on log2-scale, P-value <0.05) [42]. Further, the DON-inducing agmatine medium causes a 4.5 to 7.8 fold (log2) decrease in expression of the core cluster genes compared to glutamine enriched medium while the NPS is significantly up-regulated (7.1 fold on log2-scale, P-value <0.05) (Figure 5B) [55]. The co-expression of the genes and the conservation of the cluster in *C. heterostrophus* suggest a functional, but yet not described gene cluster.

### Ortholog analysis gives hints towards gene cluster evolution

Beside clusters that are conserved only in distantly related fungi, we detected three clusters that are unique for the species *F. graminearum* with respect to the currently available fungal genomes. The cluster C61 consists of eight genes (FGSG_10542 - FGSG_17387) comprising a PKS, a NPS, a serine hydrolase, a transcription factor and four additional genes of unknown function (Figure 6). The genes are significantly repressed (|log2-FC| >1, P-value <0.05) during C- and N- starving conditions (FG2) as well as in the FgStuA deletion mutant under secondary metabolism conditions (FG13). Six genes (FGSG_10542, FGSG_10543, FGSG_17386, FGSG_10545, FGSG_10547, FGSG_17387) exhibit an increase in expression rate during wheat infection after 64 hpi (FG19). Interestingly, *Aspergillus clavatus* is the only fungus that has a bidirectional best hit of the PKS and the NPS. The signature enzymes seem to be part of one secondary metabolism gene cluster in *A. clavatus* as they are clustered with orthologs of the serine hydrolase gene and the ABC transporter and a unique transcription factor and transporter in *A. clavatus* (Figure 6 and Table 5). However, no orthologous cluster can be found in any other fungal genome, although the signature enzymes alone are present in other species. The PKS can also be found in *Aspergillus nidulans*, *A. niger*, *A. oryzae* and *A. tereus* whereas the NPS is not present. Protein similarity suggests that an ortholog of the NPS gene is also conserved in the bacteria *Gordonia bronchialis* (37.3% similarity) and *Bacillus amyloliquefaciens* (40.3% similarity).

## Discussion

Genes which are involved in fungal secondary metabolism and in the assimilation of certain nutrients are often clustered in fungal genomes. Classical gene cluster studies focused on single gene clusters or individual genes involved in certain specific functions are now complemented by studies at the genomic level as complete fungal genome sequences and high-throughput technologies have become available. The growing number of fungal genomes available bears an opportunity to find and explore a wide range of up to now unknown fungal secondary metabolites. In *Fusarium graminearum*, a total of 51 genes involved in secondary metabolite synthesis have been predicted, exceeding the number of currently known secondary metabolites in this organism [29]–[32]. By screening the *F. graminearum* genome for spatially clustered signature and tailoring enzymes, 67 potentially functional gene clusters were identified (Table S2). Most of the clusters contain signature enzymes with unknown synthesis product and therefore constitute candidates of novel secondary metabolism pathways.

We also predicted clusters that lack a signature enzyme but exhibit an over-representation of tailoring enzymes like the cluster C09 which contains five P450 enzymes. These clusters may also be involved as modifiers in secondary metabolism pathways of other clusters or may be remnants of formerly bigger clusters. Vice versa there are also clusters containing more than one signature enzyme. C30 for example consists of a terpene synthase, a NPS and four P450 enzymes. Clusters of this high amount of secondary metabolism genes could be the result of the fusion of two clusters which act as a supercluster like recently shown in *Aspergillus fumigatus* [67]. An example is cluster C15, which comprises the 2 PKS genes (FGSG_17745 and FGSG_15980 - formerly described as *PKS3* and *PKS14*, [68]), the oxidoreductase (FGSG_15979) and the specific transcription factor (FGSG_02398), but additionally also contains other (co-regulated) genes of still unknown function including the *NPS15* gene.

The predicted clusters also include genes identified as key enzymes for biosynthesis of known compounds (Table 1). Particularly, *NPS1*, *NPS2* and *NPS6* found in three clusters are the only genes known to be involved in production of malonichrome, ferricrocin and triacetylfusarinin, respectively. The clusters may require additional genes to complete certain biosynthetic pathways. Correlation in expression profiles and the presence of over-represented promoter motifs in gene clusters provide evidence of putative pathway genes. Deletion analysis and heterologous expression of the gene clusters can help to validate them.

Our clusters defined on the basis of predicted functions of neighboring genes are comparable with gene clusters recently defined by three previously utilized approaches: ‘secondary metabolite biosynthetic (SMB) gene clusters’ [4], ‘Secondary Metabolite Unique Regions Finder (SMURF)’ [18] and ‘AntiSMASH’ [69]. All three analyses were not able to identify the already known butenolide cluster. However butenolide was detected by a generalized search of co-regulation networks [19]. SMB missed the NPS class secondary metabolite gene clusters and SMURF missed the TPS class gene clusters. A crucial difference between our SM clusters and the gene clusters found in the analyses mentioned above, results from different starting assumptions concerning signature enzymes responsible for fundamental steps in metabolite synthesis. The SMB cluster search focused only on two classes (PKSs and terpene synthase (TSs)) and SMURF used four classes of SM (PKSs, NPSs, Hybrid NPS-PKS, and prenyltransferases (DMATSs)). AntiSMASH takes more enzyme classes into consideration and is also able to detect clusters without signature enzymes. Our approach in contrast considers four types of signature enzymes (PKS, NPS, TPS and DMATS) as well as five tailoring enzyme classes (methyltransferases, acyltransferases, oxidoreductases, glycosyltransferases and cytochrome P450s) and takes transcription factors and transporter enzymes into account that might contribute to regulation and secretion of the metabolite. Overall our approach results in the most comprehensive set of potential SM clusters containing 30 clusters not found by any of the previous analyses. Vice versa SMURF and AntiSMASH detected nine and 14 clusters not found by our pipeline, respectively. Because of evidence in terms of co-expression and common promoter motifs we included in total ten additional clusters from the two prediction tools. All SMB clusters [4] were detected by our approach.

### Three gene clusters associated with an unknown metabolite are possibly involved in plant infection

Three novel gene clusters (C62, C16, C64) are expected to play important roles during plant infections, supported by remarkable expression profiles and their collection of predicted functions. All three clusters contain at least one signature enzyme as well as additional tailoring enzymes and exhibit a significant change in gene expression during plant infection. The NPS containing cluster C62 is induced after 64 hpi inside wheat coleoptiles but repressed while growing on the stem base of wheat, which hints towards a specific regulation of these genes dependent on certain plant tissues (Figure 5B). The core part of the cluster which is conserved in *Cochliobolus heterostrophus* and *Pyrenophora teres* is co-expressed. Because the NPS gene is not co-regulated with the core and orthologs of the NPS are located on separate contigs it is difficult to say whether it is part of the same biosynthesis pathway. The clusters C16 and C64 exhibit an increase in gene expression on wheat and barley as well. Like the expression profiles of the aurofusarin cluster, the profiles of the predicted clusters reach a peak after 64 to 96 hpi followed by a decrease afterwards. The *NPS9* (FGSG_10990) and the transporter gene (FGSG_10995) were mutated by Zhang et al. 2012 which resulted in reduced virulence.

The cluster C16 containing *PKS15* and a further 10 genes (Figure 3A and Table 3) is one of the most promising clusters for further analysis. *PKS15* was shown to be expressed during plant infection and has been considered as one of the strong candidates producing a metabolite of unknown function with a role in virulence [51]. However, not much information has been determined for the genes adjacent to *PKS15*: one terpenoid synthase, one cytochrome P450, one secreted protein and six further enzymes such as methyltransferase, dehydrogenase/reductase and 3-ketoacyl-acyl carrier protein reductase. Further characterization of the enzymes may provide pointers to the associated metabolite structures. No pathway-specific transcription factor is found in this cluster. Transcription seems to be controlled by other regulatory proteins affecting chromatin structure, such as a histone methyltransferase [13]. Available evidences for genes involved in a common pathway or function with *PKS15* will promote targeted research on this debatable cluster.

### Limitations of microarray data to screen fungal gene clusters

Fungal gene clusters can be difficult to detect by comparing gene expression profiles from microarray experiments alone because of the strongly diverse and selective influences on transcription of fungal genes under different conditions. The mycotoxin zearalenone (ZEN) is a good example illustrating the problem of identifying possible functions of fungal gene clusters. ZEN is found rarely in wheat or barley, but frequently in maize. Its production is favored under cold and wet conditions late in infection [70] or during storage of corn cobs [71]. In the laboratory, a temperature downshift (to 12–15°C) increases ZON production in many *F. graminearum* isolates (Jiménez et al., 1996; Ryu and Bullerman, 1999) including the sequenced strain PH-1 [72]. The available microarray data showed that ZON biosynthesis genes were not significantly expressed during infection of barley heads. In agreement with the lacking or low level expression during the infection, ZON biosynthetic genes appear to be unnecessary for infection of wheat and barley [68], [72], [73]. Thus, the ZON gene cluster would not be detected using only expression data generated on wheat and barley.

### Gene clusters possibly co-regulated due to common promoter motifs

Many gene clusters are regulated by secondary metabolism specific transcription factors [74] and global regulators [59] as well. Due to the frequent occurrence of binding sites of global regulators it is difficult to distinguish them from non-functional motifs that occur also very often in the genome. Therefore it is difficult to distinguish between random and functional motifs in a de-novo approach. Instead we focused on binding sites that are statistically over-represented on promoters of cluster genes compared to the distribution of the motifs on the whole genome. We took also promoter sequences of orthologous genes into account with the assumption that regulatory elements are conserved between species. The discovery of conserved promoter motifs as well as orthologous genes in aflatoxin-producing *Aspergillus* species [75], [76] is an example of the possible benefit of such comparisons.

The determination of specific motifs helps to identify gene clusters that may be co-regulated on the transcription level, but do not show up when expression data is analyzed. Some gene clusters, acting jointly in fungal metabolism, showed coordinated gene transcription via shared cis-regulatory elements [77], [78].

Our analysis discovered that the trichothecene mycotoxin genes have an over-representation of the conserved promoter motif 5′-TnAGGCCT-3′ (Table 4), which has been identified as TRI6 binding promoter motif for the trichothecene genes of *F. sporotrichioides* [56]. In contrast to that, Nasmith *et al.* showed that TRI6 exhibits high binding affinity to another motif which consists of repeats with the pattern GTGA (Nasmith et al., 2011). The 198 TRI6-target genes predicted by ChIP-seq experiments (Nasmith et al., 2011) contain five of the TRI-cluster genes but none of our proposed additional genes. However, the over-representation of the 5′-TnAGGCCT-3′ motif and its conservation in *F. sporotrichioides* suggests regulatory importance of the binding site by a second transcription factor.

Besides the motif of the well-studied TRI cluster we determined a putative motif in the butenolide synthesis genes, which is significantly enriched, compared to the genome-wide motif distribution and is supported by the gene expression profile of the cluster genes. Over-representation and correlation to expression data hypothesize that the predicted motif might constitute the binding site for the zinc finger transcription factor, which his located in the cluster (FGSG_08080). There are no transcription factors associated with this binding pattern so far in the Jaspar or Yeastract database [79], [80]. The palindromic motif in C02, which has been previously determined was rediscovered by our approach [55]. With the available expression data sets we could show that the putative target genes are differentially expressed in even more environmental conditions than reported before. This adds evidence to the assumption that the predicted binding site has a regulatory function.

Structures of promoter motifs can be quite diverse. Some might be shorter than a hexamer or are degenerated. Besides, such motifs can occur frequently by chance at the genomic level. Enrichment alone does not necessarily predict functionality of the motifs with high sensitivity and/or specificity. Experimental approaches like ChIP-seq or ChIP-chip experiments might be necessary to help predict binding sites.

### New additions to the trichothecene gene cluster

*Fusarium graminearum* strains produce trichothecenes, such as DON and its acetylated derivatives 3-ADON and 15-ADON, or nivalenol and acetylated derivatives, like fusarenone X. The trichothecene biosynthetic genes are well characterized in two *Fusarium* species (*F. graminearum* and *F. sporotrichioides*) and were detected at three loci on different chromosomes: a core cluster containing 12 genes, a further three genes in a mini cluster containing two genes encoding cytochrome P450 monooxygenase (*TRI1*) and acyl transferase (*TRI16*), and a single gene encoding an acetyltransferase (*TRI101*) [48], [81]–[85]. Strikingly, our analyses detected 3 additional adjacent genes, which are co-expressed with the 12 core trichothecene genes and have conserved promoter motifs. The 3 genes (FGSG_03531 (OrfA), FGSG_03530 (OrfB), and FGSG_03529) directly flanking the trichothecene core genes probably enlarge this well-known cluster. The detailed roles of the 3 genes in *F. graminearum* remains to be elucidated. Our obtained negative results of heterologous expression used for testing of the hypothesis that they are involved in deacetylation of acetyl-DON and removal of glucose from D3G do not completely exclude such roles, but make it unlikely. Potentially the transgenes were not sufficiently expressed in yeast to reveal a phenotype. Sequences similar to the putative cluster additions are present in other *Fusarium* species (e.g. *F. oxysporum*) which do not produce trichothecenes, so deacetylation of plant cell wall components seems a more likely role than deacetylation of the toxin. Also the role of OrfA, as a predicted secreted monooxygenase/tyrosinase also present in *F. oxyporum* and others, is more likely involved in the hydroxylation of monophenols and the oxidation of o-diphenols to o-quinols than in toxin biosynthesis. Also FGSG_03529 (related to glucan 1,3-beta-glucosidase, glycosyl hydrolases family 17) may play a role in producing the recently described infection structures in which trichothecene toxin synthesis occurs [86]. An important defense mechanism against the virulence factor DON in plants is its detoxification into D3G. Most likely a significant portion of the conjugate is transported by the plants to the apoplast [87]. A possible function of the predicted beta-glucosidase encoded by FGSG_03529 could be to hydrolyse DON-glucoside and to reactivate the fungal toxin. Yet, no experimental evidence for D3G hydrolytic activity of yeast expressing FGSG_03529 was observed, which also could be caused by lack of expression.

### Horizontal gene cluster transfer

Horizontal gene transfer is an evolutionary mechanism for fungi to gain new genetic material. Whereas the exchange between kingdoms including the interaction between fungi and their hosts is mostly limited to single genes [88], evidence of whole gene cluster transfers between fungi could be observed for example between *Fusarium* and *Aspergillus* [63] or *Botrytis* [89]. In *F. graminearum*, our analysis of orthologous clusters revealed 38 predicted clusters to be conserved at least partially in species outside the *Fusarium* phylum. Two of these clusters have no orthologs in the other *Fusaria*. One explanation for this observation could be that the respective cluster was present in a common ancestor and due to mutations the genes got lost individually. However in the case of the *PKS23* cluster (C47), which can be found exclusively in *F. graminearum* and the *Botrytis fuckeliana* strains B05.01 and T4, we found evidence for horizontal gene inheritance between the three species. The comparison of GC ratios of the orthologous clusters and the genomes supports the hypothesis that the cluster was transferred into the *Botrytis* lineage. In fact the GC ratios of both cluster orthologs are similar to the average ratio of *F. graminearum*, but differ significantly from the whole genome ORF GC ratio of *Botrytis*.

Although the GC ratio of the clusters fits the average ORF GC ratio of *F. graminearum*, it is unlikely that the cluster originates from that organism. There is no sequence identity between the neighboring genes of the cluster in *F. graminearum* and the genes adjacent to the *PKS23* gene in *F. pseudograminearum*, which is the only orthologous gene of the cluster in this species. Moreover, the orthologs of the *F. graminearum* cluster neighboring genes constitute a collinear region on a different scaffold compared to *PKS23* in *F. pseudograminearum*. The cluster in *B. fuckeliana* B05.01 and T4 both contain an additional collinear P450 gene that does not exist in the *Fusaria*, but GC ratio of the P450 gene is considerably higher than the average of *Botrytis*. The same holds for the additional NPS-like gene, which is unique for the B05.01 strain. The results favor the hypothesis that the original cluster was present in an unknown ancestor has at least seven genes, all present in *B. fuckeliana* B05.01, but retained only partially in T4 and *F. graminearum*. Because of the different cluster sizes in *F. graminearum* and *Botrytis*, the collinear flanking region in *F. pseudograminearum* and the difference in GC ratios, we assume that the donor organism is related to *Fusarium*.

The average GC ratios of the genomes *Cochliobolus heterostrophus*, *Pyrenophora teres* and *F. graminearum* are very similar; therefore it is more difficult to determine hints of HGT between the species based on GC ratios of cluster orthologs. Significant differences in GC ratios of orthologs of the predicted NPS clusters C62 and the host genomes could only be determined in *C. heterostrophus*, where also a reverse transcriptase could be found adjacent to the cluster. Both evidences hint towards an insertion event of the genes.

### Ortholog analysis gives hints towards evolution of gene cluster

Unique clusters in *F. graminearum* suggest sources for an exclusive metabolite that might be beneficial to the lifestyle specific to the fungus. Cluster C61 cannot be found in another fungus except *A. clavatus* where orthologs of four cluster genes, including the two signature enzymes and one neighboring gene, respectively, putatively also form a cluster. Other *Aspergilli* like *A. nidulans* or *A. tereus* contain a putative ortholog of the PKS, *Claviceps purpurea* and the bacterium *Bacillus amyloliquefaciens* contain an orthologous NPS. However, there is no other organism that contains both signature enzymes in terms of a bidirectional best hit, but *F. graminearum* and *A. clavatus*. It is likely that orthologs of the respective signature enzymes act in a different secondary metabolism pathway. The NPS ortholog in *B. amyloliquefaciens* for example, is part of the iturin A biosynthetic cluster [90] and the PKS in *A. tereus* seems to be part of a cluster with a second neighboring PKS gene. Mutations and genome reorganizations might be the driving force behind the re-shuffling and deletion of pathway genes and the creation of putatively novel metabolic products.

## Conclusions

Our analyses predict 67 secondary metabolite clusters in *F. graminearum*, reconfirming and enlarging known SM gene clusters. The 20 new clusters supported by at least one type of additional evidence are primary targets for future experiments. In particular, three gene clusters (C16, C62, and C64) are likely to have important roles involved in plant infections. Our results show that the applied methods are suited to explore secondary metabolite gene clusters in fungal genomes and give aid to select targets for further experimental studies to discover so far unknown products and/or biological functions. Ortholog analysis gives insight into the rise and fate of genes and gene clusters and therefore shed light on the host-pathogen evolution and the involved horizontal gene cluster transfer.

## Methods

### Screening the genome for secondary metabolite clusters

We used InterProScan [91] to determine functional domains and to characterize proteins. After that, putative gene clusters are calculated by scanning for local accumulations (at least three seed genes, allowing one gap) of signature and tailoring enzymes as well as transcription factors and transporters (Table S1) on the superscaffolds. The results were compared to the output of AntiSMASH [69] and SMURF [18] afterwards and adjusted manually. The statistical significance of the gene clusters to be enriched for functions associated with secondary metabolism was obtained by applying Fisher's Exact Test [35] for the four gene classes signature enzyme, tailoring enzyme, transcription factor and transporter. Resulting p-values were multiplied and corrected for multiple testing using Benjamini-Hochberg procedure [92]. In case of p<0.05 clusters are seen as significantly enriched for functions in secondary metabolism.

### Sequence data

*F. graminearum* genome data and annotation used are based on FGDB version 3.2 and the corresponding Pedant database [32]. All further genomic and proteomic data used for ortholog analysis is based on Pedant databases represented in SIMAP [65], [93] and listed in Table S3.

### Expression data

Expression data were obtained from PlexDB [37] (Table 2). All selected data sets were based on the *F. graminearum* Affymetrix gene chip [38], which was designed on the assembly version 1 and preliminary CDS annotations. In order to get expression values for the latest annotation version (3.2) we used Blast to map the probes onto the ORF-sequences, whereas only hits with 100% identity were accepted. All ambiguous probe set to ORF hits were filtered. For normalization expression data and summarization of probe-sets we used the RMA implementation of the affy R-package [94].

For the determination of significantly differentially expressed clusters we used the limma R-package [95]. P-value adjustment for multiple testing has been performed in calculating false discovery rates (FDR) using Benjamini-Hochberg procedure [92]. Genes with an absolute fold change (FC) above two with p-value below 0.05 are seen as differentially expressed. We define a predicted cluster as differentially expressed when more than 60% of the genes are significant differentially expressed. Comparisons have been performed between case and control conditions. In case of time series without control experiment, the first time point of the measurement has been taken as reference.

### Co-expression clusters

We used five time-series experiments to determine chromosomally clustered genes with correlated gene expression profile. The mean Pearson correlation coefficient (R) was used as a measure of similarity of expression profiles. For each experiment, we determined an R cutoff (R_min) as the 95^th^ percentile of 1000 Rs of randomly sampled sets of three genes. In a sliding window approach, we regard three neighboring genes as a co-expression seed when the mean R of their expression profile is above R_min and at least two genes show a significant change in their gene expression profile between two growth conditions (absolute FC above two, P-value <0.05). Seeds were extended by calculating Rs of upstream and downstream genes. Genes with R> R_min are added successively to the seed, allowing one non-correlating gene in between.

### Determination of cluster specific cis-regulatory motifs

We identified significant transcription factor binding sites by applying de- novo tools and database driven methods followed by a statistical test. In order to identify new conserved sequence motifs we utilized Meme [96], Weeder [97] and Phylocon [98] on the set of cluster promoter sequences. Additionally, we scanned for known binding sites by aligning the matrices stored in the TRANSFAC-db [99]. We defined the promoter of a gene as the 5′ intergenic sequence with a maximum of 1 kb of upstream nucleotides. As search space for the de- novo algorithms, we also included the promoter sequences of orthologous genes. All computed de-novo motifs and the matrices of the TRANSFAC-db were used as query for a genome wide promoter scan on *F.graminearum*. We assessed the significance of determined sequence motifs by applying Fisher's exact test [35], taking the occurrence of a motif on cluster promoters as well as its distribution on the genome into account. To correct for multiple testing, the resulting p-values have been adjusted using the Bonferroni procedure [100], [101]. We regard sequence motifs with a p-value below 0.01 which are present on at least eighty percent of cluster promoters as significantly over-represented for the specific cluster.

### Search for orthologous clusters

A subset of the SIMAP protein similarity database [65] was used to determine orthologous cluster genes in other species. Proteins of 181 publicly available fungal genomes, 150 bacterial reference genomes and the proteins of *Arabidopsis thaliana* were defined as search space (Table S3). All protein hits that constitute a bidirectional best hit between *F. graminearum* and the target organism with an e-value below 1e-04 and at least 50% hit overlap of the query and target amino acid sequences were taken into account. The gene order in orthologous clusters is often not conserved, thus strict collinearity is often not an adequate criterion to determine chromosomal aggregations of bidirectional best cluster hits. Therefore we selected bidirectional best protein hits that have a gene distance to each other on the target scaffold below twice the extent of the gene range of the query cluster in *F. graminearum*. To respect that some genome assemblies consist of thousands of small contigs, we also allowed a split of the cluster on more than one contig/scaffold in case the minimum aggregation of orthologs on a contig is three or above. We consider a cluster to be conserved when at least 50% of the genes in the cluster are found.

### Identification of transposable elements

Determination of transposable elements has been done by aligning the Repbase library [102] on the genomes with the help of RepeatMasker [103].

### Construction of expression vector for FGSG_03529

The gene FGSG_03529 (“related to glucan 1,3-beta-glucosidase”) contains one intron. To obtain the cDNA the two exons were amplified separately with primers containing an overlap to the other exon, to allow reconstruction of a full length ORF by fusion PCR. The primers contained the restriction sites SacI (upstream of ATG, italcs) and XhoI (C-terminal behind the stop-codon, italics) for cloning.

The primers used were FGSG_03529-SacATG: CAA GAG CTC AAC AAA ATG AAG TTT TTC AGT ACT CTC, FGSG_03529-fusion_SacATG: GCG GGG ACG GCC TTG ACG AGT GTG TTG CAG TCA GAG GCA G, FGSG_03529-C-Xho: TAT CTC GAG TTA CTT AGC AAG TAA GGC TGA AG, FGSG_03529-fusion_Xho: CTG CCT CTG ACT GCA ACA CAC TCG TCAAGGCCGTC. The resulting product of the fusion PCR was digested with SacI and XhoI and cloned into the yeast expression vector pGW830 cut with the same enzymes. This vector is essentially pYES2 (http://tools.lifetechnologies.com/content/sfs/vectors/pyes2_map.pdf) with *HIS3* replacing *URA3* as the selection marker. The structure of the expression vector (designated pCS19), with the FGSG_03529 ORF cloned behind the strong inducible *GAL1* promoter was confirmed by DNA sequencing. It was transformed into the toxin sensitive yeast strain YZGA515 (relevant genotype: pdr5,10,15 ayt1; [60]). Two independent transformants were tested for glucosidase activity with D3G.

### Construction of expression vector for FGSG_03530

The gene FGSG_03530 (OrfB, “hypothetical protein similar to acetylesterase”) also contains one intron. The ORF was reconstructed as described before by fusion PCR using the following primers: OrfB-BamATG: CAA GGA TCC AAC AAA ATG AAA TTC TCT GCC ATT G, OrfB-fusion_Bam: CAG TGA TGT GAT AAT AAT CGC AGT TCC ACC TGC T, OrfB-C-Xho: TAT CTC GAG TTA CCT ATT CTT GCC CAG TTC, OrfB-fusion_Xho: GAA GGC AGC AGG TGG AAC TGC GAT TAT TAT CAC ATC ACT CAC. The ORF was cloned into pGW830 using the restriction enzymes BamHI (italics, incorporated at the N-terminus) and XhoI. The resulting plasmid, pCS17, was also verified by sequencing and transformed into yeast using a standard Li-transformation protocol.

### *In vivo* testing for glucosidase or carboxylesterase activity

Transformants were tested for activity by growing the strains over night in induction medium (SC-HIS with 2% galactose as carbon source). For the *in vivo* assays dense overnight cultures were supplemented with the substrates to be tested. For the glucosidase assay D3G was added to a final concentration of 5 mg/L. For carboxylesterase activity tests either 3-ADON (final concentration: 10 mg/L) or 15-ADON (2.5 mg/L final, since 15-acetyl-DON is more toxic) were used. Samples of 200 µL were taken at time 0 and after 20 and 40 hours incubation (180 rpm, 30°C). The samples were combined with 200 µL acetonitrile in Eppendorf tubes and centrifuged for 10 min at 14.000 rpm. The resulting supernatant was transferred to HPLC vials, dried down with a gentle stream of nitrogen, re-dissolved in the same amount of 20% aqueous acetonitrile and used for determination of liberated DON.

Samples were measured on an 1100 series HPLC system (Agilent Technologies, Waldbronn, Germany) coupled to a QTrap LC-MS/MS system (AB Sciex, Foster City, USA). The method was based on [104] with slight modifications. A Zorbax Eclipse XDB-C8 column (150×4.6 mm, 5 µm particle size, Agilent Technologies) equipped with a 4×3 mm C18 security guard cartridge (Phenomenex, Aschaffenburg, Germany) was used for chromatographic separation. The eluents were composed of methanol water (A: 20∶80, v:v; B: 90∶10, v:v) and contained 5 mM ammonium acetate. The initial conditions of 0% B were held for 2 min, followed by a linear gradient up to 100% B within the next 2 min, a holding period of 3 min and column equilibration with the starting conditions of 0% B until the end of the run at 9 min. The flow rate was 1 mL/min and the injection volume was 40 µL. The achieved limit of quantification for DON was 15 µg/L, whereas it was 50 µg/L for D3G, 15 µg/L 3-ADON and 50 µg/L 15-ADON. While a minor DON background (corresponding to less than 2% hydrolysis in the stock solution) was observed at time 0 for all toxins, this background did not change significantly over time.

## Supporting Information

Table S1 **Secondary metabolite genes.** Predicted signature enzymes, tailoring enzymes, transcription factors and transporters used for prediction of secondary metabolism gene clusters. (Table of signature enzymes contains references to enzyme nomenclature applied in *Wiemann et al*. 2013 suppl. table 4).
(XLSX)

Table S2 **Predicted secondary metabolite clusters.** Overview of all 67 predicted clusters. Columns Metabolite and Reference indicate overlaps to clusters of known metabolites or previously defined putative clusters in Ma et al. 2010 or Zhang et al. 2012. Functional composition in terms of signature and tailoring enzymes and additional proteins is specified in the respective columns. Additional evidence of co-regulation during plant infection (Expression Profile Correlation), significant up- or down- regulation (Differential Expression), conservation of genes in other *Fusarium* species or outside the *Fusarium* clade (orthologs) as well as over-representation of putative regulatory promoter motifs are also listed.
(XLSX)

Table S3 **List of genomes used for ortholog analysis.** Extract of SIMAP protein similarity database used for the ortholog analysis of predicted gene clusters, listing species/strain data and corresponding Pedant database. Considered search space consists of 181 publicly available fungal genomes, 150 bacterial reference genomes and the proteins of *Arabidopsis thaliana*.
(XLSX)
